# Learnings from Racialized Adolescents and Young Adults with Lived Experiences of Cancer: “It’s Okay to Critique the System That Claims to Save Us”

**DOI:** 10.3390/curroncol31020081

**Published:** 2024-02-16

**Authors:** Tiffany T. Hill, Ian R. Cooper, Param K. Gill, Ada J. Okonkwo-Dappa, Cheryl A. Heykoop

**Affiliations:** Anew Research Collaborative, Royal Roads University, Victoria, BC V9B 5Y2, Canada; icooper@royalroads.ca (I.R.C.); param.7gill@royalroads.ca (P.K.G.); ada.okonkwo@royalroads.ca (A.J.O.-D.); cheryl.1heykoop@royalroads.ca (C.A.H.)

**Keywords:** adolescents and young adults, racialized, systemic and structural racism, system change

## Abstract

Interest in AYA cancer care has increased globally over the recent past; however, most of this work disproportionately represents white, heterosexual, middle-income, educated, and able-bodied people. There is recognition in the literature that cancer care systems are not structured nor designed to adequately serve people of colour or other equity-denied groups, and the structural racism in the system prevents prevention, treatment, and delivery of care. This work seeks to examine structural racism and the ways that it permeates into the lived experiences of AYAs in their cancer care. This article represents the first phase of an 18-month, patient-oriented, Participatory Action Research project focused on cancer care for racialized AYAs that is situated within a broader program of research focused on transforming cancer care for AYAs. Semi-structured interviews were completed with 18 AYAs who self-identify as racialized, have lived experiences with cancer, and have received treatment in Canada. Following participant review of their transcripts, the transcripts were de-identified, and then coded by three separate authors. Five main themes were identified using thematic analysis, including the need to feel supported through experiences with (in)fertility, be heard and not dismissed, advocate for self and have others advocate for you, be in community, and resist compliance.

## 1. Introduction

How contentious would it be to start with transparency? To start by narrating the ways our identities and experiences meet or part. To begin with who we are and how it might influence what we offer, as opposed to how impartial we can or should be. In our work together, we (the authors) are shaped by our experiences of cancer, both as young adults living with it and as care takers tending to our loved ones who experience it. We come from lands and waters far beyond where we have settled yet came to settle so differently: everything from belonging to the Nigerian, Indian, and Philippine diasporas, to being decedents of European conquerors. We bring with us learnings from the academy and the health care system while acknowledging the ways these institutions uphold our exclusion and oppression [1]. We leverage our privileges and grapple with what this means for our responsibilities to our communities, our kin, or what research might typically call our anonymous participants. Together, we gather to understand what is possible and to start anew. Given these intersections, it is fitting for our research to seek to better understand the lived experiences of racialized adolescents and young adults (AYAs) with cancer, in what is now known as Canada.

In this article, we begin offering context to our inquiry, exploring its importance and relevance in oncology. We illustrate our research process including how who we are may shape how we come to understand and interpret this work. Then, we highlight themes from our research to discuss what we learned from racialized AYAs with lived experiences of cancer and contextualize what they have said to what we understand from the literature. Themes include the need for AYAs to feel supported through experiences with (in)fertility; be heard and not dismissed; advocate for themselves and have others advocate for them; be in community; and resist compliance. To close, we share how this work will inform future work, and how it already has shaped programming and research for and with AYAs in British Columbia (BC) and Canada more widely.

## 2. Context

Over the last two decades, interest in cancer care for AYAs (people diagnosed with cancer between the ages of 15 and 39) has gained momentum globally [2,3] and nationally [4,5]. Yet most AYA-focused studies disproportionately represent white, heterosexual, middle-income, educated, and able-bodied populations [6]. Beyond this dominant narrative, there is acknowledgement that cancer care systems are not designed or structured to serve people of colour nor other equity-denied groups [7]. Instead, systems impose profound barriers to care [8], do not meet needs or concerns [9], and are founded on structural racism, which affects prevention, treatment, and delivery of care [10]. This spans across identities of ethnicity and racialization [11,12,13,14,15] and holds true for youth and adolescents [16]. Further driving disparities in care is the lack of published research conducted with racialized people from countries outside the global Western world, due to both the unwillingness of mainstream journals to publish this work, but also the high cost of “open access” journals [17].

While unsurprising given how “we have inherited and promulgated a long-standing culture of inequality” [10], our research is committed to refusal. That is, we refuse to conduct research that upholds whiteness as the highest standard of evidence [18] and we refuse to engage in research that only narrates the damage and pain of racialized communities [19]. Thus, we examine structural racism and the ways in which it suffuses in every aspect of our lives and in the lives of participants in this study, and in particular, the ways it seeps into the lived experiences of cancer.

## 3. Methodology

Our five-year patient-oriented Participatory Action Research [20,21,22,23] program is focused on working in partnership with AYAs and cancer care allies—health care professionals, decision-makers, families, researchers, and community organizations—to better understand and transform young adult cancer care research, policy, and practice across Canada. Within this program of research, we are working towards understanding the lived experiences, needs, and priorities of AYAs with diverse, intersecting identities, and broadening the research currently existing in AYA cancer care and support. This article speaks to the first phase of an 18-month initiative exploring racialized AYA cancer care experiences funded by the Canadian Institute of Health Research. Through this project, we wanted to explore how racialized young adults experience cancer care along the cancer care continuum, including their lived experiences, needs, and priorities. In doing so, we sought to explore how cancer care and support for racialized AYAs could be improved and how to mitigate systemic barriers.

Between March and August of 2023, we engaged with 18 AYAs who self-identify as racialized, have lived experiences with cancer, and have received treatment in Canada in a one-hour semi-structured interview online. We have intentionally chosen not to provide socio demographic information that we deem irrelevant to the purposes of this study, especially as studies show continued concern over the harm of collecting this information [24], and it is ineffective and inaccurate in capturing the nuance and intersections of participant identities. This study is not concerned with being generalizable, and instead is focused on learning from the lived experiences of the racialized AYAs that were involved in this study. In combination with our own efforts, recruitment included partnering with community support organizations, including Young Adult Cancer Canada (YACC), Inspire Health Supportive Cancer Care, and Callanish, to promote the project. Prior to the interview, each young adult completed a screening questionnaire to explore eligibility for the study and seek informed consent. The interview began with a brief introduction, review of consent, and an overview of the research project, and then explored the interview questions. The interviews were conducted by T.H. and audio-recorded via Zoom and then transcribed using Otter.ai. The transcripts were cleaned by A.O. to remove any personally identifiable data. Transcripts were then sent to each participant to review to validate the content of their interviews and allow them to provide any additional context or clarification [25]. Authors T.H. and I.C. developed a code book from the interviews to support data analysis and the interviews were coded individually by three authors, T.H., I.C., and one of A.O. or P.G. [26]. The code book development and interview coding were completed using the web-based coding software dedoose (Version 9.0).

The process of analysis involved the following: reviewing transcripts for salient messages, grouping these messages based on commonality, and deriving themes from these groupings [27]. From there, we developed our research themes and subthemes that included further detail on the message behind the theme, including verbatim quotes from participants. Collaborative data analysis provided us with the opportunity to look at the transcripts from different perspectives and views that we all bring with us from our lived experiences. Three out of five authors involved in the data analysis and writing of this work identify themselves as belonging to racialized communities. This may have influenced their ability to catch nuances that may not be easily identifiable. Two researchers have had personal experience navigating cancer care, as either a patient themselves or as a caregiver. This benefited in grasping the intricacies of the complex emotions that come with going through the medical system as a patient. In addition, one of the researchers is an internationally trained physician with palliative experience and another researcher is studying to be a physician. Both bring a lens that is unique in the context. The COREQ standardized reporting tool for qualitative research was followed where applicable and appropriate and is attached as Supplementary File S1 with this manuscript [28].

## 4. Discussion and Recommendations

Given our methodological approach, we value participants in the study as co-theorists; that is, we hold their own understanding of their lived experience as valuable as we do scholarship written about their lives. Thus, in this section, we offer five themes important to their lived experiences of cancer and as they intersect with their experiences as people of colour. Within each theme, we offer a discussion and recommendation. Although differently structured than traditional research papers, we have chosen this structure in consultation with those who participated in this study, making the paper more accessible, readable, and a better representation of the lived experiences of racialized AYAs in this study.

### 4.1. The Need to Feel Supported through Experiences with (In)Fertility

Although many national guidelines highlight the importance of fertility in cancer care, especially for AYAs [4,29], research has shown that concerns around fertility are infrequently discussed in the cancer care literature and by health professionals [20]. As such, it is unsurprising that we heard from AYAs that many health care workers serving in the cancer care system and fertility clinics are unprepared and ill-equipped to support AYAs who are both newly diagnosed with cancer and navigating the new reality of their (in)fertility. AYAs in this study shared that this is especially prominent through the medical and emotional process of fertility preservation. One AYA shared, “*I needed a nurse. I didn’t need a doctor, I needed someone to sit next to me, someone to inject me, someone to not be like ‘take this home and do it yourself’*”. AYAs highlighted the “*lack of oncology competency*” found within fertility clinics, one person sharing that “*I don’t feel like anyone I interacted with really understood the psychology of someone who just received a cancer diagnosis or was able to really help me go through that while also going through the other*”.

In Canada, equitable access to fertility preservation information was identified as a need amongst First Nations, Inuit, and Métis communities [30] and previous research has shown that white patients are more likely than non-white patients to seek fertility preservation [31]. While access to fertility preservation was highlighted by AYAs in the study as a need, finding support through the process was closely tied to their ability to access fertility preservation. That is, without support, even if AYAs were given the option of fertility preservation, they did not engage in it because many felt so scared and overwhelmed with the process that they were deterred from pursuing it altogether, nor did they have the financial capacity to engage in the process. Further to this, support, particularly with fertility, varies widely and is mostly limited to those with most privilege, or in other words, those not at the intersections of marginalized identities [32]. This is undoubtedly supported by the AYAs in this study, especially by those who shared their challenges about talking to their family about fertility; they most readily identified the lack of cultural understanding held by cancer care systems. One person shared, “*You don’t talk about fertility in my culture. You don’t talk about eggs and sperm. You don’t even talk about your period. I didn’t really feel equipped to have a conversation with my family who were also just coping. They were not able to be that support*”. For those who found support related to fertility outside of their homes, there was a feeling of isolation and what they described as pity. One AYA shared, “*Through infertility, I didn’t want to talk about what I was experiencing, because I knew nobody got it. So why bother talking about it. When I went to support groups, they were mostly white. And if I talked about these types of issues, it would certainly be “Oh, you poor thing”. And because of that, I can’t talk about it. You become very, very isolated*”.

In addition to racial disparities in discussions regarding fertility preservation, men-identifying patients are more than twice as likely to have health care providers discuss fertility preservation with them [33]. Yet, most AYAs in the study who raised concerns about their (in)fertility as it relates to their cancer were people who primarily identified as women. They described that the short window of time before treatment following a diagnosis was particularly challenging, leaving many to feel unable to make thorough and thoughtful decisions on their own, especially given the detrimental consequences these decisions could have on their futures.

As oncofertility programs for AYAs are being developed and funded throughout Canada (and globally), it is integral that fertility clinics and medical professionals have the knowledge and skills to better support AYAs diagnosed with cancer and to understand and respond to the diverse oncofertility needs and factors affecting AYAs’ access to oncofertility services based on their intersectional identities. Further, there is an urgent need for oncofertility programs and supports to understand cultural nuance and to develop culturally relevant information and supports for AYAs, families, and health care providers to discuss oncofertility. Further, there is an equal need for consistent financial support for AYAs seeking oncofertility services.

### 4.2. The Need to Be Heard and Not Dismissed

For many years, the health care system has been trying to shift away from its paternalistic roots and become more patient-centred. Despite this, research has demonstrated and continues to demonstrate that health care providers are more verbally dominant and less patient-centred with racialized compared to white patients [34,35]. Consistent with this, AYAs in this study identified that the system and individual providers routinely discount their experiences and symptoms. This dismissal was also exacerbated by the active upholding of power differentials, which are specifically directed towards the intersectional identities of participants and in turn put the burden of receiving adequate care on the patient. Almost every participant highlighted their experiences with being dismissed by health care providers both throughout the story of their initial diagnosis, treatment, or subsequent diagnoses. For instance, one participant shared that, “*For me, the worst, absolute worst part of the journey was getting to the diagnosis. I’m sure you hear that a lot. But as a young adult, we are never taken seriously*”.

The AYAs who participated in this study recognized that their intersectional identities were intricately related to the way in which they were being dismissed. Specifically, some recognized that their race and gender identities were distinctly involved in the way that they were being dismissed. For example, one person expressed “*I do feel, though, that if I had been a 60 year-old white man, I would not have had the whole, he’s just anxious or he’s just hysterical...I also think that race may have played a little bit of a factor there as well, just because I’ve seen studies on how white woman’s pain versus non-white woman’s pain is taken*”.

The AYAs who participated in this study also regularly described the feeling of being more than just dismissed but being gaslit by providers within the system. One AYA describes that their providers are “*just shooting darts in the dark. And I’m the one getting hit, it gets and it’s so like that level of, that level of like dismissing your reality, but not just dismissing it, they’re not just saying that it doesn’t matter*”. The participant goes on to highlight how their own symptoms and reality are turned against them: “*The symptoms don’t matter. They’re saying that it’s your fault, somehow that there’s something wrong with you or that like you’re imagining things or that you know, like there’s like nothing to do because everything should be okay with you now, because like, your blood tests are fine, everything’s fine. You should be okay*”.

In contrast, when AYAs felt heard and seen by health care providers, they found it had strong positive impacts on them personally, their families, and their care. One participant reflected this by highlighting an experience with a chemotherapy nurse: “*She actually talked to me, and she was like, oh no, you’re so young...we had a conversation about it...that was the first time that I was seen...I matter, who I am matters*”. Participants highlighted throughout the interviews that when they were not dismissed by their health care providers, they felt as though they were more than just a number and felt like a human being, seen for who they are. A participant highlights how rare and impactful feeling heard and seen was: “*It almost felt like someone like breaking character...she ended up starting to cry with us...it’s not someone who’s simply just going in and out of the rooms. Like my dad was there, it was the first time I’ve seen him cry*”. One AYA completes their thoughts on this by stating, “*I’m not this alien, because for the longest time I did, I felt like I was just alien...and just by her talking to me and having a conversation with me and staying with me for a few minutes. It made me feel like I was heard*”.

Consistent with the AYAs involved in this study, previous research demonstrates that racialized patients consistently feel that they have better relationships with their health care providers if they share that provider’s identity [36,37]. Given the significant positive impacts that AYAs describe when they feel heard and seen by health care providers, it is critical that health care providers listen to, and take seriously, the lived experiences, perspectives, and needs of AYAs. Further, to minimize further harm to AYAs, including racialized AYAs and AYAs with diverse, intersectional identities, it is vital that health care providers do not dismiss the concerns and needs of AYAs and seek to actively respond.

### 4.3. The Need to Advocate for Yourself and Have Others Advocate for You

AYAs described that advocating for themselves throughout their experience of engaging with the cancer care system was prominent and required, but not necessarily something they anticipated, wanted, or were prepared to do. AYAs highlighted how critical it was for them to advocate for themselves, especially in instances related to interactions with their health care providers. Specifically, they were challenged with advocating to feel heard and seen as it related to their presenting symptoms. One person shared, “*I had to keep advocating, advocating and finally he did an MRI. It turned out that I had a tumor that was pressed up against my sciatic nerve. When he got the results, he was like, ‘Oh, well, you have a reason to be in pain.’ And I’m like, I’ve been telling you that for how many months?*” Another person shared, “*I shouldn’t have to have to fight you for my body, for my peace of mind*” and they went on and explained, “*This person on the other end trying to convince you of something, but not taking in all the other factors in which that you’re speaking to. Which is this need of then having to advocate for that because this person doesn’t understand what you might go through, what you’ve seen others and your family live through*”. Many AYAs shared that they wished they had advocated sooner or were more adamant than they had been. AYAs experiences were that being a person of colour made it more difficult to advocate for their needs and to have their needs listened to, yet alone met.

Another way AYAs advocated for themselves was to learn about, get involved in, or join spaces of support. AYAs shared how inaccessible and unrelatable many support groups and activities were for those experiencing cancer. One person shared, “*I just felt like I was a detective trying to find as much as I could*”. Other AYAs noticed that the support groups that they fought to be in were the same spaces to teach them how to advocate for themselves. It has been known in the literature for almost four decades that patients who are taught how to advocate for themselves in health care contexts are better able to influence physicians and be actively involved in their care decisions [38]. Although many AYAs described the requirement for self-advocacy, they also wanted others to advocate for them, particularly in discussions and decision-making with their health care teams. One person shared their desire for all those experiencing cancer to have what they called ‘*bodyguards*’. They said that “*seeing my friends asking those hard questions*” really empowered them to do the same. The literature has shown that in addition to learning to advocate for themselves, social support plays a role, and for African American respondents in one study, the presence of support from male and female friends was positively associated with optimism in their care [39].

AYAs also highlighted their need to have designated workers to advocate for them within the system; those who were given advocates narrated positive experiences. For example, one person shared, “*I just go to my advocate, who then has a more personal relationship with you, she’s going to advocate with you more because she actually knows, even sees you*”. In addition, AYAs asked for advocates who could relate and understand them culturally. Other AYAs mentioned that although this role is important, the role is still tied to and controlled by the system. One person said their patient advocate “*only has so much power, right? And she’s not gonna bite the hand that feeds her*”. A recent review article highlights the importance of having systems that have patient navigators and advocates which can break down barriers to equitable access and appropriate care coordination with specific benefits to racialized patients [40]. When asked what advice AYAs would give to other young people recently diagnosed with cancer and navigating the cancer care system, almost unanimously, their responses were to advocate for yourself. Others added sentiments including “*Do your research and just take it one day at time*” or “*If you feel that you’re being ignored...ask for someone to speak to who will listen to you. Because you can’t ignore your own needs, and hope that you’ll get better*”.

In the broader advocacy context, it is still routinely evident that racialized patients’ voices are missing from the debate on how to address structural issues and barriers to improving clinician–patient relationships that could reduce inequalities in care [35]. It is important to understand the social positioning of various communities on the dominant discourse of power in our society [41]. If people in power who are making the policies, priorities, and structures of organizations and programs do not see the perspective of those who are on the “lower end” of the social positioning spectrum, this leads to the creation of the inequitable policies that build our system.

As AYA-specific programs are being developed across Canada and globally, it is imperative that AYAs are equipped with the knowledge and skills to advocate for themselves and that they have access and are supported to engage with advocates, navigators, friends, and family who can advocate alongside them. Further, there is an urgent need for care providers to engage AYAs as partners in their own care. When advocates or navigators are supporting AYAs, it is critical that AYAs have the opportunity to work with navigators who look like them and understand their lived experiences. Considering the needs expressed by AYAs, the role of an AYA advocate or navigator is integral. The AYA advocate or navigator would be the first point of contact for AYAs navigating the cancer care system and their primary job would be to support and advocate for the needs and priorities of AYAs. Recognizing the structural and systemic inequities in the cancer care system, AYA advocates or navigators would require training and capacity in cultural awareness and specific cultural knowledge, would have strong relationships and connections with culturally appropriate supports, and would ideally be representative of the diverse AYAs they support and serve.

### 4.4. The Need to Be in Community

Research suggests that AYAs with cancer benefit from participating in support groups given the community they are able to build with one another [29]. This is echoed in this study, where most AYAs discussed their need to be in community; in fact, their sense of togetherness extended beyond their experiences of relating to one another, but also into the ways they look out for each other. For instance, one AYA shared how important it was for them to vet the study we were conducting. They did this by participating themselves before encouraging others to do so, checking for relational approaches and non-extractive ways of engaging in research. While building community is of importance, AYAs in the study emphasized how challenging it was to find spaces where they did not feel othered based on their age and their race. For instance, in trying to relate to others in support groups, one AYA shared, “*We’re just at such different stages of our lives that they have way different things to worry about and be upset about than me. So, it was like I don’t belong*”. This is substantiated by a study in the literature in which women of colour with breast cancer highlighted how unwelcome they feel accessing support groups, where membership is predominantly white and resources are centered around a “‘model’—white, middle-class patient” [20]. Further, AYAs frequently note that they need AYA-specific programs and support groups as it is difficult to relate to others at a different life stage or age and who may have different lived experiences and care needs [42]. 

Similarly, many AYAs highlighted how dominantly underrepresented they felt in support groups, but also in waiting rooms, hospitals, and offices. While holding the weight of describing these moments of noticing when they were the “only one” or “*one of few*”, one person shared, “*I basically requested a therapist with a similar background to me. I found that when I spoke to her…I was never invalidated for what I was saying or thinking. And she was above the baseline expectation of not having that bias. She was able to really bring my own context to be a strength*”. Evidence suggests that having a doctor from same race or ethnicity improves a patient’s health care experience and decreases implicit bias from the physician [43,44,45]. Yet, even with one-fifth of the population in Canada identifying as people of colour [46], there is limited diversity representation among physicians. In addition, in one study, many racialized survivors of breast cancer believed that race and language detrimentally affected the quality of care they received [47]. As reflected in this study, AYAs felt seen by people who looked like them or could relate to their experience, but they also signaled at their providers’ ability to understand the critical role community and family play in their lives. For providers to truly understand how to care for racialized AYAs and care about their decisions and needs, AYAs cannot be looked at without thinking of their family and community contexts [48].

As the number of AYAs diagnosed with cancer continues to rise, it is critical that AYAs interface with and connect with individuals who look like them and understand them, their families, and communities as they navigate cancer care systems. Representation in cancer care systems is an integral step to begin to establish/re-establish trust within a broken system that has done little to address structural and systemic racism—but it is not the only step. For racialized AYAs and health care providers to thrive in this system built to be oppressive, the system must shift to better serve them, think of them, center them. 

### 4.5. The Need to Resist Compliance

AYAs claimed that throughout their experience with the cancer care system, they often found themselves embodying what one person called the role of the model minority. They described this as, “*I’ve just tried to be as good a patient as I could, so that it would be easy for the health care provider. I wanted their experience to be really good. And I don’t know where that came from. You know, model minority? Like you don’t try and make waves. And as much as I hate the model minority myth. It’s like, did I just embody that? I don’t know, or was I just so unwilling to be in my own body?*” AYAs told stories of acting and making decisions around their care simply out of feeling the pressure to comply with what their health care providers wanted or deemed best. Most AYAs felt unable to contest or question their care given the power dynamics between them and their providers [34,35,49]. When AYAs offered experiences where they did question their care, they described how often this would end in being treated as insignificant, titled unintelligent, or even being refused treatment. One person shared, “*And I remember thinking to myself, this is what happens when you don’t agree with and go with the white man right?*”.

Given such experiences of racism and discrimination, many AYAs still insisted that those they encountered in the health care system are not “*excluding people on purpose*” or that they “*don’t mean any malice*”. Some AYAs indicated that they were the lucky ones and did not experience overt racism and rationalized this as they are not “*dark enough*” or that they do not speak with an accent. Research tends to be familiar with this attitude, given that one study described racialized people as less likely to describe discrimination in their oncology care and predominantly expressing gratitude for their oncology providers even with lower satisfaction with care or medical error occurring, instead attributing blame to health care systems [47]. Other AYAs in this study shared this more nuanced reflection, sharing the following:

*“We pick up on it subconsciously, what people feel for us, and their attitudes towards us. And when that’s screaming, you’re in danger from the people in places that are providing your care and saving your life. In order to silence that voice … It’s like telling your brain, let’s cut this out, stop telling me stop sounding the alarms that we’re in danger. I’m getting the help I need, I need to be grateful. But at the end of the day, you’re never going to know what the care you needed would have looked like without that hate, without those alarms”*

In essence, AYAs have a strong urgency to resist compliance and to not be silenced. The system and those who uphold it, however, hold the kind of power that convinces racialized AYAs to question if refusal will be what kills them or, in the case of cancer, keeps them from living. While some AYAs in this study held gratitude for their health care providers and even to the systems that house them, what is clear from this study is that racialized AYAs are equally as eager to be critical of the oppressive systems that govern their lives and to shape what is possible for their futures.

## 5. Conclusions

Racialized AYAs with lived experiences of cancer have clearly identified their needs and priorities. They have identified their desires to be supported, listened to, and advocated for. They have signaled the importance of community and have encouraged a continued critique of the health care and cancer care systems that (try to, at least sometimes) serve them. While they narrate their gratitude to the providers who work tirelessly to attend to their care and save their lives, they contend with the structural and systemic racism that they cannot seem to escape. As part of an 18-month initiative, the themes discussed in this article will inform the next phase of our research, in which racialized AYAs who were interviewed will participate in a 6-week creative series. In this series, AYAs will engage in arts-based and creative activities to more deeply explore, understand, and make meaning of their lived experiences of cancer and identify tangible changes to better support AYAs navigating cancer care. Further, this study informs ongoing research within Anew to better understand, inform, and shape cancer care for all AYAs with diverse, intersectional identities and lived experiences. Although at times insufferable, as researchers and scholars, we have a responsibility to not just change the cancer care system, but to turn it on its head; and to do so for and with the AYAs we learn from. Our hope with this article is that it informs how we begin this pursuit, in the smallest of increments or the biggest of leaps.

## Data Availability

The data are available on request from the corresponding author due to privacy reasons.

## Supplementary Materials

The following supporting information can be downloaded at: https://www.mdpi.com/1718-7729/31/2/81/s1, File S1: COREQ (COnsolidated criteria for REporting Qualitative research) Checklist.
